# Efficacy and Long-Term Outcomes of Intra-Articular Autologous Micro-Fragmented Adipose Tissue in Individuals with Glenohumeral Osteoarthritis: A 36-Month Follow-Up Study

**DOI:** 10.3390/jpm13091309

**Published:** 2023-08-26

**Authors:** Simone Natali, Daniele Screpis, Edoardo Patania, Luca De Berardinis, Andrea Benoni, Gianluca Piovan, Venanzio Iacono, Bruno Magnan, Antonio Pompilio Gigante, Claudio Zorzi

**Affiliations:** 1 Department of Orthopaedics, IRCCS Ospedale Sacro Cuore Don Calabria, 37024 Negrar, Italy; simone.natali@sacrocuore.it (S.N.); daniele.screpis@sacrocuore.it (D.S.); gianlucapiovan@sacrocuore.it (G.P.); venanzio.iacono@sacrocuore.it (V.I.); claudio.zorzi@sacrocuore.it (C.Z.); 2 Department of Orthopaedics and Trauma Surgery, University of Verona, Piazzale A. Stefani 1, 37136 Verona, Italy; edoardopatania@gmail.com (E.P.); andrea.benoni8@gmail.com (A.B.); bruno.magnan@univr.it (B.M.); 3 Clinical Orthopaedics, Department of Clinical and Molecular Sciences, Università Politecnica delle Marche, 60020 Ancona, Italy; a.p.gigante@staff.univpm.it

**Keywords:** osteoarthritis, shoulder, adipose-derived stem cells, mesenchymal stem cells, lipoaspirate, orthobiologics, biologic treatment, regenerative medicine, retrospective study

## Abstract

Background: Glenohumeral osteoarthritis (GOA) is associated with disabling shoulder pain that affects everyday life. Its management comprises various treatment approaches, both conservative and surgical. Regenerative medicine has gained a major role in the conservative treatment of osteoarthritis. Intra-articular injection of adipose-derived mesenchymal stem cells (ADMSCs) is a widely used regenerative medicine approach. The aim of this retrospective study was to report the safety and clinical outcomes of intra-articular injection of ADMSCs in patients with GOA over 36-months. Methods: This retrospective observational study involved patients with chronic shoulder pain resistant to standard conservative treatment and a diagnosis of concentric GOA, who received an intra-articular injection of autologous micro-fragmented adipose tissue (μFAT). The values of the Constant–Murley score (CMS), the visual analog scale (VAS), and the simple shoulder test (SST), collected at baseline and at 12, 24, and 36 months, were analyzed to assess treatment efficacy. The single assessment numeric evaluation (SANE) was used to rate patient satisfaction. The Friedman test was used to compare observations of CMS, VAS, and SST values repeated on the same subjects. The significance threshold was set at 0.05. Results: The participants were 65 patients with a mean age of 54.19 years and a nearly equal gender distribution. Most had mild concentric GOA classified as Samilson–Prieto grade 1. The mean follow-up duration was 44.25 months. The postoperative clinical scores showed significant improvement. At 36 months, the CMS was 84.60, the VAS score was 3.34, and the SST score was 10.15 (all *p* < 0.0001). The SANE score at 36 months indicated that 54 patients (83.08%) were completely satisfied with the treatment. Conclusion: ADMSC treatment exerted favorable effects on the clinical outcomes of patients with GOA, providing pain relief and improving shoulder function. Our data support its use as a conservative treatment option for osteoarthritis.

## 1. Introduction

### 1.1. Epidemiology

Osteoarthritis (OA) is a highly complex multifactorial condition, where cartilage, subchondral bone, and the synovial membrane play a critical role in disease development [1]. Alterations in the subchondral bone are closely associated with degeneration of the articular cartilage [2]. In particular, as the cartilage degeneration progresses, the affected joints show increasing bone volume as well as trabecular thickness [3,4]. An additional feature is the stiffening of the bone structure, which is capable of undermining the ability of bone to absorb impact loads, resulting in elevated stress on and damage to cartilage [3,4,5]. With a projected occurrence of 10% among males and 18% among females aged above 60, OA stands as the most commonly observed musculoskeletal condition across the globe. The glenohumeral joint is the third most commonly affected major joint after the knee and the hip [6]. Yet, our knowledge of the progression of arthritic changes in the shoulder is limited. A cohort study conducted to monitor the advance of radiographic changes due to OA in the knee found an annual disease progression rate of 2.8% over a 15-year period [7]. Whether the shoulder follows a similar course to other joints is still unclear. However, there are currently no interventions capable of reversing or slowing down the natural progression of early OA.

### 1.2. Etiology

Glenohumeral osteoarthritis (GOA) can loosely be categorized into primary and secondary categories. Primary GOA accounts for the overwhelming majority of cases (approximately 90%) and typically affects individuals aged 60 years or older. It commonly involves degeneration of the articular cartilage, abnormally dense subchondral bone, development of osteophytes, and erosion of the posterior glenoid, with consequent posterior displacement of the humeral head [8]. Notably, such changes do not appear to be related to injury or to surgery, even though factors such as obesity, shoulder overuse injuries, job tasks involving repetitive use of the upper limbs, participation in overhead sports, and a history of trauma or dislocation have all been implicated in the development of shoulder OA [8,9].

Secondary GOA is more common in younger patients [9,10]. Other potential, albeit less common, causes of secondary GOA include inflammatory arthropathy, radiofrequency and/or thermal capsulorrhaphy, and the sequelae of joint infection.

### 1.3. Conservative, Surgical and Orthobiologic Treatment of Shoulder Osteoarthritis

Before undertaking surgical management of GOA, a non-operative treatment approach aiming to mitigate pain, enhance function, and where possible minimize disease progression should be considered [10,11]. General non-surgical approaches, such as modifications of everyday tasks and physical exercise, medicinal treatment, therapeutic physical interventions, and intra-articular injections, are the mainstays of non-operative management of GOA [10,11]. In addition to their potential to alleviate symptoms, these approaches offer the key advantages of cost-effectiveness and minimal risk. Then, as the disease and symptoms progress, surgical treatment provides an effective cure. However, whereas current arthroplasty techniques have consistently achieved successful outcomes in elderly patients with GOA who do not respond to non-operative treatment, the outlook is less favorable for younger patients, as prosthetic replacement often fails to meet their greater activity demands and functional expectations [12]. The possible occurrence of complications and adverse outcomes and the limited lifespan of prosthetic devices have prompted the search for novel, non-operative treatment options for this subgroup of patients in order to manage their condition until they meet the indications for arthroplasty [12]. Building upon the early success of autologous orthobiologic therapies in joints such as the knee, we set out to assess the role of similar approaches in the glenohumeral joint [7,11]. Orthobiologic therapies hold significant promise and opportunity. For instance, a variety of formulations derived from the density separation (centrifugation) of blood, e.g., platelet-rich plasma (PRP), and bone marrow, e.g., bone marrow aspirate concentrate, are successfully being used due to their ability to modulate inflammation [13]. Preparations based on cells derived from adipose tissue have also been providing encouraging results [14,15]. The wide range of cytokines, anti-inflammatory factors, and bioactive molecules found in these preparations serve as crucial regulators of the complex healing process that characterizes the joint microenvironment, suggesting that they could be applied to treat degenerative joint conditions [16,17]. Nevertheless, several questions concerning the utilization, safety, and efficacy of orthobiologics still need to be addressed.

In the past decade, the use of orthobiologics in shoulder surgery has rapidly expanded. Commonly employed biologics in such patients include progenitor cells, growth factors, PRP, and biological matrices. The potential benefits of incorporating biological augmentation in traditional shoulder surgery protocols include minimal invasiveness, enhanced healing capacity, and expedited recovery. However, their wider use is currently hampered by cost and by limited evidence of their long-term effectiveness. Moreover, the literature regarding the use and efficacy of biologics in shoulder surgery is quite variable in terms of indications, type of product, processing methods, and mode of administration. Notably, some studies have reported encouraging results for isolated biologic treatment or biologic-enhanced surgery, whereas others have demonstrated no consistent advantages.

Products based on PRP and mesenchymal stem cells (MSCs) have undergone a rapid surge in popularity after they were reported to improve knee function and pain [18]. However, the findings of magnetic resonance imaging studies are conflicting, since some have failed to document tissue regeneration or an increase in cartilage thickness [19,20], whereas others have described high levels of glycosaminoglycans in designated regions of the treated articulation [21]. MSCs act as trophic agents by sensing their surroundings [22] and releasing soluble factors and regulatory molecules encapsulated in extracellular vesicles, such as microRNAs (miRNAs), which are collectively known as the “secretome” [23]. These elements effectively restrain inflammation, impede tissue fibrosis and apoptosis, and activate intrinsic progenitor cells in the tissue [24].

The role of MSCs as “guardians of inflammation” [25] has gradually emerged from extensive research conducted over several decades. This work has consistently demonstrated that in most conditions MSCs only appear temporarily in injured tissues. During their brief presence, MSCs engage in communication with injured cells to limit tissue destruction or enhance repair through a variety of mechanisms. These mechanisms include (a) the activation of genes that regulate excessive inflammatory and immune reactions, (b) the creation of a supportive environment to enhance the proliferation and differentiation of endogenous stem/progenitor cells, and (c) the transfer of vesicular components containing mitochondria and miRNAs.

Adipose-derived mesenchymal stem cells (ADMSCs) possess significant multipotency, particularly in the chondrogenic lineage [26]. Adipose tissue, due to its abundance in the body and ease of collection compared with other tissues such as bone marrow, has been recognized for its potential in treating joint degeneration [27]. Micro-fragmented adipose tissue (μFAT), one of several adipose-tissue-derived products created to date, is obtained through the action of gentle mechanical forces followed by removal of proinflammatory blood and oil remnants without additives, enzymes, or centrifugation [27]. Encouragingly, recent studies have demonstrated significant clinical improvement following μFAT injection to treat knee and ankle OA [28,29,30,31,32].

Given the increasing use of ADMSCs in the conservative treatment of OA, the aim of this study was to examinate functional outcomes, pain, and satisfaction in a cohort of patients with GOA treated with intra-articular injection of autologous micro-fragmented adipose tissue over a 36-month follow-up period. 

## 2. Materials and Methods

### 2.1. Study Design

This is simple retrospective design with one group [33] that was carried out at a single institution. Informed consent, encompassing the utilization of medical and personal data, was obtained and endorsed by patients prior to treatment. All the surgical procedures respected the principles of the Declaration of Helsinki as revised in 2014.

The study was approved by the Ethics Committee of Verona and Rovigo, Italy (protocol no. 61386-19 September 2018). 

We searched the institutional database for the records of the patients who received an intra-articular injection of autologous μFAT in the shoulder joint from October 2018 to June 2020 at IRCSS Sacro Cuore-Don Calabria Hospital (Negrar di Valpolicella, Italy) and had a follow-up pereiod of at least 36 months.

### 2.2. Inclusion and Exclusion Criteria

Inclusion criteria were as follows: patients with a history of at least 6 months of chronic shoulder pain resistant to conservative treatment, e.g., physical therapy, activity modification, non-steroidal anti-inflammatory drugs, corticosteroids, PRP, or hyaluronic acid injection, and a clinical and radiological diagnosis of concentric GOA.

Exclusion criteria were acute or chronic rotator cuff tears, acute tendonitis or bursitis, adhesive capsulitis, impingement syndrome, chronic subacromial pain, eccentric GOA, and severe concentric or eccentric GOA with a recommendation of shoulder replacement. 

All procedures were performed by a single surgeon (S.N.).

### 2.3. Surgical Technique and Postoperative Care

Participants were positioned in supine position on a table in a dedicated room. Employing aseptic and sterile conditions, a small incision was executed in the abdominal region, below the umbilicus under local anesthesia. This incision facilitated the insertion of a 17 G blunt cannula that was connected to a Luer lock syringe with a volume capacity of 60 cc. Subsequently, patients underwent percutaneous administration of a solution composed of 500 mL of saline, 50 mL of lidocaine at a concentration of 2%, and 1 mL of epinephrine at a dilution of 1:1000 into the subcutaneous adipose tissue of the abdominal region. Following a 10 min interval, approximately 60 mL of adipose tissue was hand extracted using a 13 G blunt cannula connected to the syringe. The acquired lipoaspirate was then subjected to processing through the Lipogems^®^ system (Lipogems International S.p.a., Milano, Italy) as recommended by the manufacturer [34]. This system encompasses a disposable apparatus comprising a transparent cylindrical container housing stainless steel spheres. Preceding the introduction of the lipoaspirate, the container was filled with saline. Subsequent to this, mechanical agitation was applied to the lipoaspirate, thereby facilitating its fragmentation. Post-fragmentation, the container was subjected to saline irrigation in order to purge impurities, following which, a total of 7 mL of the derived μFAT product was extracted and transferred into a syringe. Under ultrasound guidance, the μFAT was then rapidly injected intra-articularly through a posterior approach and utilizing a 22 G needle. Subsequent to the injection, a series of passive range of motion exercises were administered. Participants were released from the medical facility approximately 2 to 3 h post-procedure and were provided with post-operative instructions. Initiating from the day of the operation, mobilization of the shoulder joint and muscle strengthening exercises were initiated and sustained for a minimum of 2 weeks. Patients were advised to incorporate cold therapy and rest for at least 24 h. Gradual resumption of mild activities and sports were permitted based on individual tolerance levels. The adipose tissue donor site was subject to medication every 3 days. An abdominal binder was worn for a duration of 15 days, after which sutures were removed.

### 2.4. Preoperative Evaluation and Follow-Up

Before the surgical procedure, we collected the following patient data: gender, age, body mass index (BMI), American Society of Anesthesiologists (ASA) class, affected side, and whether arthroscopy had been performed before the procedure.

We examined the X-rays taken for GOA diagnosis and graded the stage of arthrosis according to Samilson–Prieto grade [35,36]: 0 (normal joint); 1, mild (osteophytes < 3 mm on the humeral head); 2, moderate (osteophytes ranging from 3 to 7 mm involving either the humeral head or the glenoid); and 3, severe (osteophytes > 7 mm with/without joint incongruity).

We applied a range of clinical scoring systems to assess the efficacy of the ADMSC treatment.

The Constant–Murley score (CMS) was used for the preoperative and postoperative assessment of shoulder pain (up to 15 points), activities of daily living (up to 20 points), range of movement (up to 40 points), and strength (up to 25 points). The maximum score (100 points) is given for an absence of symptoms and good health, the lowest score is 0 [37,38,39,40]. Preoperative and postoperative shoulder pain intensity was also assessed using the visual analog scale (VAS), which is depicted as a linear continuum featuring uniformly spaced numbers ranging from 0 (no pain) to 10 (pain of the highest imaginable intensity) [41].

The simple shoulder test (SST), a rapid and effective 12-item questionnaire requiring affirmative or negative responses, was also employed to assess shoulder pain and function before and after the procedure [37,42,43]. Satisfaction with the procedure was measured at 36 months using a method based on the literature, the single assessment numeric evaluation (SANE), where patients are asked to answer the question: “How would you rate your shoulder today as a percentage of normal on a 0–100% scale with 100% representing normal” [37,42,44]. Ratings ≥ 80% were classified as completely satisfactory outcomes (score, 1), those ranging from 60% to 80% were considered as reflecting adequate treatment (score, 2); and those ≤60% were considered as unsatisfactory outcomes (0).

### 2.5. Statistical Analysis

All analyses were conducted using Excel (Microsoft (version 16.75.2), Redmond, WA, USA) with the XLSTAT resource pack (XLSTAT-Premium, Addinsoft Inc., New York, NY, USA). The Shapiro–Wilk test was performed to assess whether the data showed a nonparametric distribution. The calculated mean values were also reported for all continuous data. Percentage frequencies were used for qualitative variables. The baseline and postoperative clinical scores were compared using the nonparametric Friedman test, which is used for repeated measures analysis, to determine differences in the Constant-Murley score (CMS), the visual analog scale (VAS), and the simple shoulder test (SST) at baseline and at 12, 24, and 36 month follow-ups. A *p*-value < 0.05 was considered significant.

## 3. Results

The participants were 65 patients, 33 men (50.77%) and 32 women (49.23%), with a mean age of 54.19 years (SD, 9.63). Their age ranged from 34 to 73 years. The right shoulder was affected in 35 patients (53.85%) and the left in 30 (46.15%). The mean BMI was 25.00 kg/m^2^ (SD, 3.44), range 18.79–36.58. There were 47 patients (72.31%) with ASA 1 (good health), 17 (26.15%) with ASA 2 (mild systemic disease), and a single patient (1.54%) with ASA 3 (severe systemic disease).

As regards to the radiological stage using Samilson–Prieto grading, 53 patients (81.54%) were rated as grade 1 (minimal or no osteoarthritis changes), 7 patients (10.77%) as class 2 (moderate osteoarthritis changes), and 5 patients (7.69%) as class 3 (severe osteoarthritis changes). Four patients (6.15%) had undergone preoperative arthroscopy.

The mean follow-up duration was 44.25 months (SD, 6.04), range 36 to 58.

All these data are reported in Table 1.

All the clinical scales reflected significant improvement in all measures (Table 2). The baseline CMS value was 73.74 (SD, 12.60; range, 37.00–87.00). The follow-up values increased to 84.23 at 12 months (SD, 10.81; range, 56.00–97.00), 87.60 at 24 months (SD, 8.23; range, 66.00–98.00), and 84.60 at 36 months (SD, 10.68; range, 48.00–100.00).

Compared with the baseline score of 5.57 (SD, 1.64; range, 3.00–9.00), the postoperative VAS scores fell to 2.99 at 12 months (SD, 1.64; range, 1.00–6.00), 3.15 at 24 months (SD, 1.42, range; 1.00–6.00), and 3.34 at 36 months (SD, 2.02; range, 1.00–8.00).

The preoperative SST value of 8.19 (SD, 1.32, range, 4.00–10.00) rose to 10.06 at 12 months (SD, 1.42, range, 6.00–12.00), 10.91 at 24 months (SD, 0.91, range, 8.00–12.00), and 10.15 at 36 months (SD, 1.47; range, 6.00–12.00).

The Friedman test demonstrated statistically significant differences in CMS, VA, and SST during the entire follow-up (*p* < 0.0001).

The SANE results at 36 months were ≤60% (unsatisfactory outcome) for 5 patients (7.69%), ≥80% (complete satisfaction) for 54 patients (83.08%), and between 60% and 80% (adequate treatment) for 6 patients (9.23%).

## 4. Discussion

We designed this study to assess the effect of autologous μFAT on chronic shoulder pain and GOA. Several systematic reviews have demonstrated the safety and efficacy of stem cell therapy in patients with a variety of orthopedic conditions [45].

In particular, MSCs have the potential to facilitate cartilage tissue repair, reduce inflammation, and alleviate OA pain, providing significant improvement as assessed by several widely accepted clinical rating systems [46,47]. The three clinical scores used in our study supplied comprehensive information regarding the effect of ADMSC treatment on pain, daily activities, strength, and overall shoulder function. The CMS improved significantly at 12 months, it rose further at 24 months, then at 36 months it reverted to the values reached at 12 months. Likewise, the perception of pain, measured with the VAS, showed that the treatment induced significant pain relief throughout the first 12 months, with a minor resurgence of pain at 24 and 36 months that never reverted to the baseline values. In contrast, the SST score rose significantly at each time point and never declined.

Clearly, the improvement experienced by our patients cannot match the imporovements achieved with other procedures, especially joint replacement. For arthroplasty, Kim et al. reported a change in the mean CMS from 35.4 to 57.8 points at 24 months [48], whereas Sershon et al. described a significant reduction in the VAS pain score, from 6 to 2.1, and a significant increase in the SST score, from 1.4 to 6.2, at 2.8 years [49].

However, the results of such studies cannot be compared with ours, principally because patient characteristics determine the indication for conservative or surgical treatment. Indeed, most of the patients enrolled in our study had mild concentric GOA according to the Samilson–Prieto classification and were consequently more likely to benefit from ADMSC treatment and experience greater improvement. In contrast, patients receiving a joint replacement are typically older and have a more severe condition. The disparity between the two types of patients thus depends on the different indication for treatment. In younger patients and in those with more advanced OA, ADMSC treatment may be critical to alleviate pain and bridge the interval to joint replacement. Finally, the effectiveness of ADMSC treatment is reflected in the satisfaction of our patients, since at 36 months 54 of our 65 participants were completely satisfied with the outcome of their procedure. This finding provides novel evidence for the effectiveness of ADMSC treatment in patients with chronic shoulder pain and GOA and is in line with the earlier reports [50,51].

Our study has two main strengths: a large sample size compared with similar studies and a follow-up of at least three years. Notably, the therapeutic effects of the treatment began to decline at two years, particularly pain reduction.

Among the limitations of the study are the lack of a control group, such as a cohort receiving hyaluronic acid or corticosteroid injection, and the absence of pre- and postoperative MRI to prove cartilage repair. Furthermore, the outcomes were not subjected to stratified analysis based on age, BMI, or the OA. A stratification based on the degree of OA was not performed, consequently, clinical outcomes could not be assessed in patients with severe grades of Samilson–Prieto classification. Lastly, the postoperative phase omitted the surveillance of patient activities aimed at mitigating issues such as excessive joint utilization. Another drawback is the fact that only four patients underwent preoperative arthroscopy, since the scan would have provided useful information both for differential diagnosis and for the assessment of therapeutic effects. In addition, the extensive saline irrigation performed during arthroscopy may ameliorate local inflammation [52].

All these limitations should be taken into account when interpreting the findings of our research, and future studies should address these aspects to enhance the understanding of ADMSC treatment in shoulder conditions.

## 5. Conclusions

This retrospective observational study of the safety and efficacy of autologous μFAT in patients with chronic shoulder pain and GOA documented significant improvements in clinical outcomes as measured by the CMS, the VAS, and the SST. The changes compared with baseline were statistically significant at 12, 24, and 36 months. The SANE demonstrated that most patients were completely satisfied.

These findings support the safety and efficacy of autologous μFAT as a treatment for chronic shoulder pain and GOA. Notably, in younger patients and in those with more advanced OA, conservative treatment with ADMSCs may be a valuable tool to alleviate pain and bridge the interval to joint replacement.

Further studies are warranted to validate these results and explore longer-term outcomes.

## Figures and Tables

**Table 1:** Preoperative and perioperative data.

Variable	Patients
Number	65
Age, mean (SD) [range]	54.19 (9.63) [34.00–73.00]
Gender	
Male (%)	33 (50.77)
Female (%)	32 (49.23)
Side:	
Right (%)	35 (53.85)
Left (%)	30 (46.15)
BMI (kg/m^2^), mean (SD) [range]	25.00 (3.44) [18.79–36.58]
ASA class	
ASA 1 (%)	47 (72.31)
ASA 2 (%)	17 (26.15)
ASA 3 (%)	1 (1.54)
Samilson–Prieto classification	
Grade 1 (%)	53 (81.54)
Grade 2 (%)	7 (10.77)
Grade 3 (%)	5 (7.69)
Preoperative arthroscopy	
Yes (%)	4 (6.15)
No (%)	61 (93.85)
Follow-up (months), mean (SD) [range]	44.25 (6.04) [36.00–58.00]

SD: standard deviation; BMI: body mass index; ASA: American Society of Anesthesiology.

**Table 2:** CMS, VAS, and SST values before the procedure and at 12, 24, and 36 months. SANE ratings at 36 months.

	Values	*p*-Value
CMS		<0.0001
Baseline, mean (SD) [range]	73.74 (12.60) [37.00–87.00]	
12-month follow-up, mean (SD) [range]	84.23 (10.81) [56.00–97.00]	
24-month follow-up, mean (SD) [range]	87.60 (8.23) [66.00–98.00]	
36-month follow-up, mean (SD) [range]	84.60 (10.68) [48.00–100.00]	
VAS		<0.0001
Baseline, mean (SD) [range]	5.57 (1.64) [3.00–9.00]	
12-month follow-up, mean (SD) [range]	2.99 (1.64) [1.00–6.00]	
24-month follow-up, mean (SD) [range]	3.15 (1.42) [1.00–6.00]	
36-month follow-up, mean (SD) [range]	3.34 (2.02) [1.00–8.00]	
SST		<0.0001
Baseline, mean (SD) [range]	8.19 (1.32) [4.00–10.00]	
12-month follow-up, mean (SD) [range]	10.06 (1.42) [6.00–12.00]	
24-month follow-up, mean (SD) [range]	10.91 (0.91) [8.00–12.00]	
36-month follow-up, mean (SD) [range]	10.15 (1.47) [6.00–12.00]	
SANE		
36-month follow-up,		
0 (%)	5 (7.69)	
1 (%)	54 (83.08)	
2 (%)	6 (9.23)	

CMS: Constant–Murley Score; VAS: Visual Analogue Scale; SST: Simple Shoulder Test; SANE: Single Assessment Numeric Evaluation.

## Data Availability

The data presented in this study are available on request from the corresponding author.
